# From maternal glucocorticoid and thyroid hormones to epigenetic regulation of offspring gene expression: An experimental study in a wild bird species

**DOI:** 10.1111/eva.13598

**Published:** 2023-10-03

**Authors:** Mikaela Hukkanen, Bin‐Yan Hsu, Nina Cossin‐Sevrin, Mélanie Crombecque, Axelle Delaunay, Lotta Hollmen, Riina Kaukonen, Mikko Konki, Riikka Lund, Coline Marciau, Antoine Stier, Suvi Ruuskanen

**Affiliations:** ^1^ Institute for Molecular Medicine Finland University of Helsinki Helsinki Finland; ^2^ Department of Biology University of Turku Turku Finland; ^3^ Institut des Sciences de l'Evolution de Montpellier (ISEM) Université de Montpellier, CNRS, IRD, EPHE Montpellier France; ^4^ Turku Bioscience Centre University of Turku and Åbo Akademi University Turku Finland; ^5^ Turku Doctoral Programme of Molecular Medicine University of Turku Turku Finland; ^6^ Institute for Marine and Antarctic Studies University of Tasmania Hobart Tasmania Australia; ^7^ Institut Pluridisciplinaire Hubert Curien, UMR 7178 University of Strasbourg, CNRS Strasbourg France; ^8^ Department of Biological and Environmental Science University of Jyväskylä Jyväskylä Finland

**Keywords:** epigenetics, hormone, maternal effects, methylation, *Parus major*, prenatal

## Abstract

Offspring phenotype at birth is determined by its genotype and the prenatal environment including exposure to maternal hormones. Variation in both maternal glucocorticoids and thyroid hormones can affect offspring phenotype, but the underlying molecular mechanisms, especially those contributing to long‐lasting effects, remain unclear. Epigenetic changes (such as DNA methylation) have been postulated as mediators of long‐lasting effects of early‐life environment. In this study, we determined the effects of elevated prenatal glucocorticoid and thyroid hormones on handling stress response (breath rate) as well as DNA methylation and gene expression of glucocorticoid receptor (GR) and thyroid hormone receptor (THR) in great tits (*Parus major*). Eggs were injected before incubation onset with corticosterone (the main avian glucocorticoid) and/or thyroid hormones (thyroxine and triiodothyronine) to simulate variation in maternal hormone deposition. Breath rate during handling and gene expression of GR and THR were evaluated 14 days after hatching. Methylation status of GR and THR genes was analyzed from the longitudinal blood cells sampled 7 and 14 days after hatching, as well as the following autumn. Elevated prenatal corticosterone level significantly increased the breath rate during handling, indicating an enhanced metabolic stress response. Prenatal corticosterone manipulation had CpG‐site‐specific effects on DNA methylation at the GR putative promoter region, while it did not significantly affect GR gene expression. GR expression was negatively associated with earlier hatching date and chick size. THR methylation or expression did not exhibit any significant relationship with the hormonal treatments or the examined covariates, suggesting that TH signaling may be more robust due to its crucial role in development. This study provides some support to the hypothesis suggesting that maternal corticosterone may influence offspring metabolic stress response via epigenetic alterations, yet their possible adaptive role in optimizing offspring phenotype to the prevailing conditions, context‐dependency, and the underlying molecular interplay needs further research.

## 1 INTRODUCTION

Maternal effects occur when parental phenotype directly affects the offspring phenotype (Bernardo, 1996). These effects may persist throughout one's lifetime and even to subsequent generations (Bernardo, 1996; Mousseau & Fox, 1998). Maternal effects may be adaptive when mothers' experience of the environment is transmitted to the next generation (e.g., via molecular markers, hormones, resources, or care), and when this increases offspring fitness (Love & Williams, 2008; Mousseau & Fox, 1998; Weber et al., 2018; Yin et al., 2019; Zhang et al., 2020). Hormones are one of the main mechanisms of maternal effects because they affect various traits by altering gene expression and cellular functions (Groothuis et al., 2005, 2019; Podmokła et al., 2018). Since environmental factors such as food abundance alter maternal hormone production and transfer to the offspring, maternal hormones may program the offspring to better cope with the prevailing environmental conditions (Groothuis et al., 2019). However, maternal hormonal effects may also be due to mere physiological constraints, and their adaptive role remains debated (Groothuis et al., 2005; Sánchez‐Tójar et al., 2020; Zhang et al., 2020).

Maternal hormonal effects have been widely explored in oviparous species, such as birds. Since the embryo develops outside of the mother's body, maternally transferred hormones can be measured and manipulated uncoupled from the mother's physiology (Groothuis et al., 2019). Maternally derived steroid hormones have been found to have long‐lasting or even transgenerational effects on postnatal phenotype and fitness‐related traits such as growth and reproduction (Groothuis et al., 2005; Podmokła et al., 2018). In birds, both glucocorticoids (GCs) and thyroid hormones (THs) are transferred from the mother's blood to the egg yolk and can lead to both transient and/or long‐lasting phenotypic changes in the offspring (Hayward & Wingfield, 2004; Ruuskanen & Hsu, 2018; Schoech et al., 2011). Yet, the role of prenatal GCs and THs has been less studied compared to, for example, maternal testosterone (Bentz et al., 2021; Groothuis et al., 2019), while GCs and THs are involved in behavior, metabolism, and stress response, and therefore prenatal GCs and THs are expected to play major roles during offspring development (Ahmed et al., 2016; Groothuis et al., 2019; Hayward & Wingfield, 2004; Love & Williams, 2008). For instance, *in ovo* corticosterone manipulations have been shown to alter the hypothalamus–pituitary–adrenal (HPA) axis response, which is crucially involved in stress physiology (Haussmann et al., 2012; Love & Williams, 2008; Tilgar et al., 2016). Great tits (*Parus major*) hatching from eggs with experimentally elevated corticosterone (CORT, main avian glucocorticoid) levels also exhibit prolonged begging behavior and increased breath rates (Tilgar et al., 2016). Breath rate is at least partly controlled by the autonomic nervous system and when measured during handling, it reflects the rise in metabolism linked to the stress response (Careau et al., 2008; Carere & van Oers, 2004; Yackle et al., 2017). Although breath rate is unlikely to be directly controlled by the HPA axis, a facilitating role of baseline GC levels on the autonomic nervous system exists (e.g., Ouyang et al., 2021). This could mediate the effect of prenatal CORT on breath rate (Tilgar et al., 2016), for example, if prenatal CORT exposure increases baseline CORT that exerts facilitating functions. Differences in behavior, metabolism, and stress response may represent different strategies to cope with environmental challenges (Carere et al., 2001; Koolhaas et al., 1999; Romero, 2004). As the most advantageous coping strategy can be dependent on the prevailing environmental conditions (e.g., food abundance, predation pressure, population density, weather unpredictability; Carere et al., 2005; 2008; Koolhaas et al., 1999), prenatal exposure to maternal hormones may be important in preparing the offspring for the expected environmental conditions after hatching to maximize fitness prospects (Groothuis et al., 2005). Yet—regardless of whether maternal hormonal effects are adaptive or not, the molecular mechanisms underlying effects on phenotype remain poorly understood (Bentz et al., 2021; Groothuis et al., 2019; Groothuis & Schwabl, 2008).

Biological functions of GCs and THs are mainly facilitated through binding to their respective receptors (Groothuis & Schwabl, 2008; Henriksen et al., 2011). Characterizing variation in receptor expression, complementary to measuring circulating levels of hormones has been recently emphasized as crucial to understand hormonal effects (e.g., Zimmer et al., 2023), and maternal hormonal influence on postnatal receptor expression could be one mechanism underlying their effects on phenotypes. The prenatal hormonal environment may affect postnatal receptor expression via effects on epigenetic regulation (i.e., mechanisms translating the information of a genotype to various phenotypes; Waddington, 1942). Epigenetic alterations may function as a tool for individuals to acclimatize to changing environmental conditions, but they may also mediate transgenerational adaptation (Guerrero‐Bosagna et al., 2018; Sepers et al., 2021; Vogt, 2021). One of the best‐studied epigenetic mechanisms is DNA methylation, which facilitates changes in gene expression, imprinting, and transposon silencing (Jaenisch & Bird, 2003; Laine et al., 2022; Vogt, 2021), and is known to be affected by age, environmental quality, and physiological condition (De Paoli‐Iseppi et al., 2019; Lindner et al., 2021; Mäkinen et al., 2022; Siller Wilks et al., 2023). Alterations in the methylation of the genes related to HPA‐axis function, especially hormone receptor genes such as glucocorticoid receptor (GR, transcribed by *NR3C1)*, have been suggested to mediate the effects of prenatal exposure to glucocorticoids (Ahmed et al., 2014; Groothuis & Schwabl, 2008; Jimeno et al., 2019; Ruiz‐Raya et al., 2023; Zimmer & Spencer, 2014). High concentrations of *in ovo* corticosterone have been found to increase offspring GR gene methylation and decrease the receptor protein expression in chicken (*Gallus gallus domesticus*) hypothalamus (Ahmed et al., 2014). The role of receptor gene methylation in mediating the effects of prenatal thyroid hormones has received less attention in previous studies: Van Herck et al. (2012) discovered that TH supplementation to chicken egg yolk increased TH concentration in the brains of chicken embryos 24 h after manipulation and altered TH membrane transporter expression and thyroid hormone receptor (THR, transcribed by *THRA* and *THRB*) expression, yet effects on methylation have not been investigated. The few studies (Ahmed et al., 2014; Van Herck et al., 2012; Zimmer & Spencer, 2014) that have addressed the epigenetics of prenatal hormonal effects by directly manipulating the egg hormone concentrations have (1) mainly focused on glucocorticoids, and (2) hormone manipulation occurred when incubation already started. Manipulations during incubation do not mimic maternal deposition and could lead to different effects since CORT and TH are likely to be at least partly metabolized by the growing embryos (CORT: Vassallo et al., 2019; TH: Ruuskanen et al., 2022). Additionally, the impact on methylation pattern has only been examined cross‐sectionally for a relatively short period after hatching so far, which precludes to evaluate the potential long‐lasting nature of epigenetic changes induced by maternal hormonal effects, knowing that human studies have shown both consistency and flexibility in methylation patterns with time within an individual (Komaki et al., 2021). Although such longitudinal approach is deeply needed to evaluate within‐individual consistency and flexibility, it restricts investigations to tissues that can be sampled repeatedly (e.g., blood cells). In this context, it is important to acknowledge that there is evidence that methylation levels can be tissue‐specific, and some data suggest that the effects of maternal hormones on methylation patterns could vary across tissues (e.g., Husby, 2020; Sepers et al., 2019).

To fill these gaps, we experimentally elevated glucocorticoid (i.e., corticosterone) and thyroid (i.e., triiodothyronine, T3 and thyroxine, T4) hormone concentrations within the natural range in wild great tit eggs before incubation onset to simulate the causal effects of maternally elevated hormones on offspring phenotype. These two hormones were selected because of their implication in energy metabolism and stress response. Breath rate in response to handling was assessed as an indirect measure of acute metabolic stress response (Carere et al., 2001; Carere & van Oers, 2004; Fucikova et al., 2009). We then investigated possible alterations in the methylation status of the glucocorticoid and thyroid hormone receptors using a longitudinal study design (blood sampling of the same individuals at days 7 and 14 post‐hatching, as well as early adulthood), and assessed gene expression patterns at a single time‐point (day 14 post‐hatch).

Considering their effect on stress response and metabolism, we predict that the prenatal glucocorticoid and thyroid hormone exposure will increase the offspring breath rate during handling (*reviewed in* Thayer et al., 2018). We also set out to examine potential changes in blood cell gene expression and DNA methylation due to prenatal hormonal treatment, which might reflect a general pattern across tissues (Ewald et al., 2014; Palma‐Gudiel et al., 2015), while acknowledging that the effects may differ and have different consequences within other tissues such as the hypothalamus–pituitary axis. Given that stress response is regulated by a negative feedback system (Cottrell & Seckl, 2009), we predict that higher prenatal exposure and possibly enhanced intrinsic GC production during early development has increased the GR gene methylation and downregulated its mRNA expression. Our predictions are also supported by an association between high baseline plasma corticosterone and low GR expression in zebra finches’ blood cells (*Taeniopygia guttata*, Jimeno et al., 2019), as well as by the fact that prenatal stress has been found mainly to decrease GR expression (*reviewed in* Cottrell & Seckl, 2009; Kapoor et al., 2006 yet see Zimmer et al., 2017). However, for the effects of TH, such directional predictions are challenging to make. To our knowledge, the only study that has assessed the effects of prenatal TH supplementation in THR expression in avian models found prenatal TH to increase THR expression during embryonic development but did not analyze postnatal expression or measure DNA methylation (Van Herck et al., 2012). Based on this, we would predict elevated THs to increase THR expression accompanied by decreased THR methylation. Furthermore, elevated levels of both thyroid hormones and glucocorticoids could lead to synergistic effects, as shown in fish and amphibian models (Brown et al., 2014; Buisine et al., 2021), and therefore a factorial design was used to detect such potential synergistic effects between prenatal TH and CORT. We predict decreased methylation patterns with age, following Gryzinska et al. (2013), who reported a decrease in global methylation in chicken blood cells between 1‐day‐old chicks and 32‐week‐old hens (yet see De Paoli‐Iseppi et al., 2019). However, directional predictions of the age‐related methylation changes in our target genes need to be approached with caution, all genomic regions may not follow the global pattern and could even exhibit opposite patterns to global trends (De Paoli‐Iseppi et al., 2019), and there may be differences in the direction of methylation changes even between CpG sites within one gene during the course of development (Siller Wilks et al., 2023).

## 2 MATERIALS AND METHODS

### 2.1 Study population

The study was conducted in a wild great tit nest box population (374 boxes in 10 plots, all within 3 km range) in Ruissalo, Southwestern Finland (60° 26’ N, 22° 10′ E). Nesting activity was monitored every 4–5 days. The nests where egg‐laying had started were visited daily until hormone manipulation was completed. The experimental protocol has been described in detail in Cossin‐Sevrin et al. (2022): this study concerns a subsample of the nests included in the larger study (Table 1).

**TABLE 1:** Number of individuals and nests by treatment group in the whole experiment and for different analyses (breath rate, methylation, gene expression).

	*In ovo* treatment				Total
	CO	CORT	CORT + TH	TH	
*N* breath rate	27 (10)	20 (10)	16 (7)	20 (9)	83 (36)
*N* methylation	10 (10)	10 (10)	9 (9)	10 (10)	39 (39)
*N* gene expression (*NR3C1/THRA*)	10/10 (10)	8/9 (9)	6/6 (6)	9/8 (9)	34 (34)

*Note*: Numbers of nests are given in brackets. Treatment groups are coded as follows: CO, control; CORT, corticosterone; CORT + TH, corticosterone and thyroid hormone combination group; TH, thyroid hormone. There were 1–3 randomly selected individuals per nest for the breath rate analysis. One randomly selected individual per nest was included in the methylation and gene expression analyses. All individuals in the gene expression analyses were included in the methylation analyses. We tried to also maximize the overlap between data on breath rate, methylation, and gene expression from the same individuals, but this was not always possible due to limited blood sample availability. In the end, 18 individuals with methylation data had also breath rate data, and 16 individuals with gene expression data had also breath rate data. There were 45 different nests in total (CO = 11, CORT = 12, CORT + TH = 10, and TH = 12).

### 2.2 Hormone manipulations

The mean final clutch size of great tit nests was 8.2 (SD = 1.83), ranging from 2 to 10. All eggs from one nest received the same treatment. The nests were assigned to four treatment groups: control (CO, *N* = 11 nests), glucocorticoid hormone supplementation (CORT, *N* = 12), thyroid hormone supplementation (TH, *N* = 12), and a combination of glucocorticoid and thyroid hormone (CORT+TH, *N* = 10). The treatments were assigned to the nests randomly, but sequentially so that all treatments would be equally distributed across the breeding season, and attention was given to geographical distribution (i.e., all treatments present in all forest plots). Egg injection started on the day of the 5th egg (as females may start to incubate before clutch completion) and every day thereafter for newly laid eggs, which ensured that no incubation had occurred at the time of the injection. The doses of TH and CORT were chosen to increase the average content in the yolks by 2SD (Podmokła et al., 2018). Each egg in the TH group was injected with a combination of 0.32 ng of T4 and 0.04 ng of T3. For the corticosterone treatment group, 0.2 ng was injected per egg. The combination group (CORT+TH) received the sum of all hormones (0.32 ng T4, 0.04 ng T3, and 0.2 ng corticosterone). Each egg received an injection of 2 μL, containing the hormone in question, dissolved in 0.1 mol/L NaOH (TH) or 99% ethanol (CORT), and diluted in 0.9% NaCl. Eggs in control clutches were injected with 2 μL of 0.9% NaCl. Injections were conducted using sterile equipment and yolk was visualized using a LED lamp from below. For more details on the injection procedure, see Cossin‐Sevrin et al. (2022). Hatching success was not affected by the hormone treatments (Cossin‐Sevrin et al., 2022).

### 2.3 Phenotypic measurements

The nests were visited daily starting 2 days before the predicted hatching to record the hatch date (=day 0). Nestlings were visited 2, 7, and 14 days after hatching for identification, phenotypic measurements (weight, wing length), and blood sampling (days 7 and 14) as described in Cossin‐Sevrin et al. (2022). Additionally, 14 days after hatching, we measured handling stress response as a proxy of different stress‐related coping strategies (Carere & van Oers, 2004; Fucikova et al., 2009) by assessing the individual's breath rate (see below) in response to handling (Carere & van Oers, 2004; Fucikova et al., 2009; Liang et al., 2018). Breath rate was measured from one to three randomly selected chicks per nest, immediately after taking each individual from the nest (before other measurements) following the protocols described in Carere and van Oers (2004) and Fucikova et al. (2009). The breath rate was measured as breast movements during a 60‐s time. The entire measurement lasted for 75 s per individual (15‐s interval × 4, 5 s in between). Breath rate was calculated as the sum of breast movements during the four intervals. Sex of the individuals was determined from 14‐day blood samples for which DNA was available using a qPCR approach adapted from Ellegren and Sheldon (1997) and Chang et al. (2008). Details are described in Cossin‐Sevrin et al. (2022).

Individuals were recaptured as juveniles (ca. 9–20 weeks after fledging) in the following autumn: 20 of the 39 individuals included in the methylation analysis were recaptured. Mist nets (with playback) were set up in seven feeding stations across the study area. Each feeding station was visited for 3 h per netting on 3 distinct days during October–November (total of 100 h of mist netting). Weight and wing length measurements, as well as blood samples, were collected for juvenile individuals applying the same workflow as above.

### 2.4 Methylation analysis

#### 2.4.1 DNA methylation: DNA extraction

DNA methylation of glucocorticoid receptor gene *NR3C1* and thyroid hormone receptor B *(THRB)* were detected by bisulfite conversion followed by pyrosequencing. For each nest, one randomly selected individual was used in the methylation analysis. DNA was extracted from the frozen blood samples of 40 great tit individuals, each of which were sampled longitudinally two or three times (Table 2). The DNA samples were stored at −80°C after extraction. DNA extraction and quality assessment are described in Cossin‐Sevrin et al. (2022).

**TABLE 2:** Number of samples/CpG sites included in the methylation analysis after quality filtering for each gene (*NR3C1*, glucocorticoid receptor; *THRB*, thyroid hormone receptor β), treatment group (CO, control; CORT, corticosterone; CORT + TH, corticosterone and thyroid hormone; TH, thyroid hormone), and age (DAH, days after hatching).

Age	CO	CORT	CORT + TH	TH	Total
NR3C1					
7 DAH	10/119	9/108	9/106	10/112	38/445
14 DAH	10/112	10/120	8/95	10/117	38/444
Juvenile	5/59	5/60	5/54	5/60	20/233
Total	25/290	24/288	22/255	25/289	96/1122
THRB					
7 DAH	10/40	10/39	8/32	10/39	38/150
14 DAH	10/39	10/40	7/28	10/40	37/147
Juvenile	5/18	4/16	5/20	5/20	19/74
Total	25/97	24/95	22/80	25/99	96/379

#### 2.4.2 DNA methylation: Bisulfite conversion

Bisulfite conversion of the DNA samples was conducted by using EpiTect Fast DNA Bisulfite Kit (Qiagen, cat. 59824) and by following the manufacturer's high concentration protocol. For each sample, 20 μL of genomic DNA (10 ng/μL) was used as a starting material. The cleaned bisulfite‐converted DNA samples were stored at 4°C, and the following PCR was conducted within 24 h.

Thyroid hormone receptors are coded by two genes: alfa and beta. The epigenetic regulation of *THRB* has previously been shown to be involved in many human phenotypes such as cancer, obesity, and aging (Joseph et al., 2007; Kim et al., 2013; Ling et al., 2010; Pawlik‐Pachucka et al., 2018; Shimi et al., 2022), and affected by exposures to, for example, thyroid hormones and environmental toxins in mice (Cho et al., 2021; Laufer et al., 2022), and thus we were interested in investigating if that is the case also in the context of avian maternal hormones, and chose *THRB* for the methylation analyses.

Chromosomes 13 (for *NR3C1*, GenBank assembly accession GCA_001522545.3) and 2 (for *THRB*, GenBank assembly accession CM003710.1) of the great tit genome (GenBank assembly accession GCA_001522545.3) were retrieved from NCBI's repository (NCBI Resource Coordinators, 2016; Yates et al., 2019). For both genes, *NR3C1* and *THRB* (transcript variant X5), a region from 1800 base pairs upstream to 100 base pairs downstream of the transcription start site was selected as the putative regulatory region as with zebra finches in a study by Jimeno et al. (2019) on *NR3C1*. Within this region, primers were designed to amplify CpG‐dinucleotide dense regions with PyroMark Assay Design Software 2.0. Sequence of the amplified fragments and their location with respect to the gene are shown in Supplementary Figure S1. Primers were validated in PCR with bisulfite‐treated samples (eight samples tested) and gel electrophoresis (1.5%, 90 V). Primer characters are shown in Table 3.

**TABLE 3:** Primer sequences used to detect the methylation status of the putative promoter regions of the glucocorticoid receptor gene (*NR3C1*) and thyroid hormone receptor gene β (*THRΒ*).

NR3C1	Gene ID:107210791	CpG (N°):16	
Primer ID	Direction	Sequence	
PCR F1	Forward	AGAAGGTAGAGTTGGAGGTAGATAG	
PCR R1	Reverse	ACCCCCCTTCTATATACCAAATTAAAA	Biotinylated
Sequencing S1	Forward	TTGTAGGGTGTTTATTTTAAGTAG	
THRΒ	Gene ID: 107215324	CpG (N°):16	
Primer ID	Direction	Sequence	
PCR F1	Forward	GGGGTGTATGTTTGTTTGTGT	
PCR R1	Reverse	TCCCCCCCCTCCCACAATCA	Biotinylated
Sequencing S1	Forward	ATTTTTGGAGTAGTAGTTAATT	

*Note*: Forward and reverse primer sequences are presented with the number of CpG sites (N°) within each sequence to analyze.

#### 2.4.3 DNA methylation: PCR

The target regions of the genes of interest were prepared for amplification by using PyroMark PCR Kit (Qiagen, cat. 978903) following the manufacturer's protocol. Bisulfite‐treated DNA (4 μL, 10 ng/μL) was added to the reaction mixture (without optional MgCl_2_). The thermal cycler (Applied Biosystems 2720) program varied according to the target gene: 15 min at 95°C, 45 cycles of 20 s at 94°C, 30 s at 56°C (*NR3C1*) or 60°C (*THRB*), 30 s at 95°C, and a final extension of 10 min at 72°C. The concentration (>20 ng/μL) of the amplified samples and the negative controls were measured with NanoDrop ND‐1000, Thermo Scientific. Samples were frozen (−20°C) after PCR, and pyrosequencing was conducted within 3 weeks.

#### 2.4.4 DNA methylation: Pyrosequencing

For pyrosequencing, (*NR3C1* and *THRΒ*), all the samples (*N* = 99 individuals, a total of 198 samples) were analyzed in five batches. All samples from the same individual at different ages were in the same batch, and the treatments were distributed as evenly as possible between the batches. One pyrosequencing run included only one gene assay (*NR3C1*/*THRΒ*).

Pyrosequencing was conducted by using PyroMark Q24 Advanced CpG Reagents (Qiagen, cat. 970922) and with PyroMark Q24 Pyrosequencing instrument (Qiagen), following the manufacturer's protocol using 15 μL of PCR product and 2 μL of Streptavidin Sepharose High‐Performance beads (GE Healthcare, cat. GE17‐5113‐01).

#### 2.4.5 DNA methylation: Quality filtering

Pyrosequencing results were first assessed in PyroMark Q24 Advanced Software (3.0.1). The pyrosequencing results included the methylation percentage and quality ranking for each CpG site (*N*_*NR3C1*_ = 16, *N*_*THRΒ*_ = 16) within each sample (*N*_Sample_ = 99, in total 1584 sites for both genes where methylation was detected). Sites where the quality was classified as “Failed” by the software were discarded (*NR3C1*: 205/1584 discarded, *THRΒ*: 703/1584 discarded). As the quality of the methylation percentages decreased toward the 3′ end of the sequence, most of the discarded “Failed” data were in the 3′ end of the analyzed sequence. To ensure no CpG sites were significantly underrepresented in the analysis after quality filtering, we discarded all CpG sites with data from less than 85 out of the total 99 samples (i.e., each CpG site had data from at least 86% of the entire sample size, for *NR3C1* 4/16 CpG sites were discarded, for *THRΒ* 12/16 CpG sites were discarded). All the methylation percentage observations with clearly deviating residuals after fitting the statistical models were considered as technical outliers and thus discarded (*NR3C1*: 3/1125 observations, DNAm% range with outliers 0%–24.5%, without outliers 0%–5.09%, i.e., >9 SD, *THRΒ* none).

After quality filtering, there were data from 12 CpG sites of 96 samples from 39 individuals for the glucocorticoid receptor gene *NR3C1* (Table 2). For *THRΒ*, there were data from four CpG sites of 96 samples of the same 39 individuals (Table 2). Sex ratios per treatment group are given in Supplementary Table S1.

### 2.5 Gene expression analysis

The expression of glucocorticoid receptor gene (*NR3C1*) and thyroid hormone receptor genes (*THRΑ* and *THRΒ*) was examined with RT‐qPCR (reverse transcription quantitative PCR) following the MIQE guidelines (Bustin et al., 2009). *THRB* could not be properly quantified (qPCR quantification cycle values >30, which may be due to an absence of expression in blood cells); thus, the final analysis included *NR3C1* and *THRA* as genes of interest and two reference genes (see below).

#### 2.5.1 Gene expression: RNA isolation and reverse transcription

RNA was successfully isolated from blood cells of 34 fourteen‐day‐old great tits with NucleoSpin RNA Plus Kit (Macherey‐Nagel). Packed blood cells (10 μL per sample) were transferred to lysis buffer and homogenized with a sterile micro‐pestle, after which the remaining steps were conducted following the manufacturer's protocol with a final elution in 50 μL of RNase‐free H_2_O. The purity and concentration of extracted RNA were measured with a spectrophotometer (ND‐1000, Thermo Scientific). Absorbance ratios 260/280 > 1.8 and 260/230 > 1.8 were considered thresholds for purity. Samples with RNA concentration less than 25 ng/μL concentration were re‐extracted from the original samples when possible. RNA integrity was validated using gel electrophoresis (E‐Gel 2%, Invitrogen) and the ribosomal RNA 18S versus 28S bands intensities. Samples not fulfilling the above‐mentioned quality criteria were discarded (6/40). Isolated RNA samples were stored at −80°C for 3 weeks before reverse transcription. For each sample, 600 ng of isolated RNA was reverse transcribed to complementary DNA (cDNA) with SensiFAST cDNA Synthesis kit (Bioline) following the manufacturer's protocol. Reverse transcribed cDNA samples were stored at 4°C and were analyzed in qPCR within a week.

#### 2.5.2 Gene expression: RT‐qPCR primers

The primers for the quantitative PCR are shown in Table 4. Primers for the great tit glucocorticoid receptor (*NR3C1*, NCBI ID: 107210791*)* were designed by Casagrande et al. (2020). Thyroid hormone receptor α (*THRΑ*, NCBI ID: 107215324) primers were designed using Primer‐BLAST (Ye et al., 2012) using the great tit genome (GenBank assembly accession GCA_001522545.3). The reference genes used in the analyses were *SDHA* (succinate dehydrogenase complex flavoprotein subunit A, NCBI ID: 107200805) and *RPL13* (ribosomal protein L13, NCBI ID: 107209800), for which the primers were designed and previously validated by Verhagen et al. (2019).

**TABLE 4:** Primer sequences and performance for the genes of interest (*NR3C1* and *THRΑ*) and reference genes (*SDHA* and *RPL13*) used in RT‐qPCR.

Target	Forward	Reverse	BP	Cq (SE)	*E* (SE)	Intraplate CV (%)	Interplate CV (%)	*R* (SE)
NR3C1	GGAATAGGTGCCAGGGATCG	TTCCAGGGCTTGAATAGCCA	102	25.58 (0.17)	94.92 (0.41)	0.67 (0.14)	0.94 (0.15)	0.97 (0.01)
THRΑ	GAGGGCTGCAAGGGTTT	CTGGTTGCGGGTGATCTT	107	25.34 (0.16)	91.92 (0.32)	0.64 (0.10)	0.82 (0.36)	0.98 (0.01)
SDHA	GGGCAATAACTCCACGGCAT	TTGTATGGCAGGTCTCTACGA	99	20.78 (0.10)	95.80 (0.30)	0.61 (0.07)	0.60 (0.08)	0.99 (0.01)
RPL13	TACTCCTTCAGCCTCTGCAC	ACAAGAAGTTTGCCCGGACT	99	18.78 (0.17)	91.08 (0.38)	0.39 (0.05)	0.58 (0.18)	1.00 (<0.01)

*Note*: Forward and reverse primer sequences are presented with expected amplicon lengths (BP). Cq is the average quantification cycle value for each gene with associated standard error (SE). *E* refers to the average amplification efficiency calculated by LinRegPCR algorithm described by Ramakers et al. (2003) with the formula: *E* = 10^*slope*^ − 1. The slope is determined with linear regression from the amplification curve. Intraplate CV (%) is the average coefficient of variation for the duplicate samples, and interplate CV (%) is the coefficient of variation for two repeated qPCR plates. Technical repeatability (*R*) for duplicate samples is also given.

Primers for qPCR were first validated using pooled cDNA from four distinct great tit individuals that were not included in the final analysis. Primer specificity and optimal annealing temperature were confirmed by ensuring each primer produced a single narrow peak in the melt curve. NT‐controls (sterile MQ‐H_2_O) and template RNA (no reverse transcription) were confirmed to show no amplification before at least five cycles after the higher Cq of the samples of interest. A two‐fold serial dilution of template cDNA from 1.5 ng to 24 ng was used to create a standard curve to evaluate primer efficiency at a wide range of starting RNA concentrations. A high‐resolution melt curve analysis was used to assess the uniformity of the amplified DNA sequences as the dissociation behavior of the double‐stranded DNA depends on the DNA sequence. Gel electrophoresis was used to ensure a single PCR product of the expected length for a random subset of samples. Reference gene stability was assessed with geNorm (Qbase+, Biogazelle, Belgium, Vandesompele et al., 2002), which calculates the stability of expression (M) for each gene. *M*_*SDHA*_ and *M*_*RPL13*_ were both below 0.7, which is the recommended upper limit for the stability value (M) of a reference gene (Vandesompele et al., 2002). The reference gene expression (Cq) did not differ between the treatment groups (ANOVA‐test: *SDHA*: *F*_3,29_ = 0.31, *p* = 0.81, *RPL13*: *F*_3,29_ = 1.12, *p* = 0.36).

#### 2.5.3 Gene expression: Quantitative PCR

The relative quantity of the reverse transcribed target cDNA was assessed using magnetic induction cycler (micPCR, Bio Molecular Systems) and SensiFAST SYBR Lo‐ROX Kit (Bioline). For each gene, samples were analyzed in two 48‐well qPCR plates. All biological samples were run as technical duplicates on the same plate. Additionally, pooled samples from four great tit samples were run in quadruplicates to serve as calibrator samples for expression normalization. Each plate also included duplicates of sterile H_2_O with no template controls and RNA samples which were not reverse transcribed as controls. For each well, 5 μL of cDNA (1.2 ng/μL) was combined with 6 μL of SensiFAST SYBR Lo‐Rox Mix, 0.18 μL forward and reverse primers (300 nM) and 0.64 μL of sterile H_2_O (*V*_tot_ = 12 μL) in strip tubes preloaded with mineral oil. Quantitative PCR was run in the magnetic induction cycler with the following program: 95°C 120 s, (95°C 5 s, 60°C 20 s) × 45.

#### 2.5.4 Gene expression: Gene expression normalization and quality filtering

Each plate was confirmed to have a single amplification peak for each primer set and NT controls were confirmed to show no amplification. Relative expression for each sample was assessed with Pfaffl method (Pfaffl, 2001) using the formula below:

$$\text{Relat}i\mathrm{ve}\;\text{gene expression}=\frac{{E_{\mathrm{GOI}}}^{∆\mathrm{Cq}\;\mathrm{GOI}\;\left(\text{calibrator}-\text{sample of interest}\right)}}{\text{Geom}.\;\text{mean}\;\left[{\left(E_{\mathrm{REF}}\right)}^{∆\mathrm{Cq}\;\mathrm{REF}\left(\;\text{calibrator}-\text{sample of interest}\right)}\right]}$$

*E* refers to the average efficiency for each gene in each plate (theoretical maximum would be perfect doubling at each PCR cycle = 2). Efficiencies were obtained from micPCR Software output, which calculates them using the LinRegPCR algorithm described by Ramakers et al. (2003) with the formula: *E* = 10^*slope*^ − 1. The slope is determined with linear regression from the amplification curve. Cq is the quantification cycle value for each sample as the number of cycles needed for the fluorescence (describing PCR product quantity) to reach a threshold set by the LinRegPCR algorithm (Ruijter et al., 2009). The calibrator Cq is the pooled sample in each run.

Relative gene expression for each individual was calculated as the mean relative expression for the technical duplicates, which was log_2_ transformed for further statistical analyses. Samples with over 30% CV between the relative expression values of the technical duplicates were discarded. Model residuals showed no outlier samples.

### 2.6 Statistical analysis

All statistical analyses were conducted in R Studio (version 4.0.3, R Core Team 2020). To examine variation in breath rate, DNA methylation, and gene expression, we used linear (mixed) models, using base R and package *lme4* (Bates et al., 2015), while type III ANOVA was calculated using the package *lmerTest* (Kuznetsova et al., 2017) and *car* (Fox & Weisberg, 2019). We inspected the normality and homogeneity of variance visually from the model residuals. *F*‐statistics (with associated degrees of freedom) and *p*‐values from type III ANOVA were calculated with the Kenward–Roger method for the mixed models (breath rate and methylation analysis). Random effect significance was calculated using likelihood ratio test by comparing models with and without the random effects. Post‐hoc comparisons were assessed with package *emmeans* (Lenth, 2022) using Tukey's multiple comparison procedure. *emmeans* was also used to calculate effect sizes of the hormone treatments. Multicollinearity of model covariates was assessed with variance inflation factor (VIF) using R package *car* (Fox & Weisberg, 2019). All variables in all models had VIFs within the range of 1–1.51, which is below 5 that is commonly considered as the threshold for multicollinearity (Petrie, 2020). We performed model diagnostics using the R package *DHARMa* (Hartig, 2022), and via visual inspection. R packages *ggplot2* (Wickham, 2016) and *ggpubr* (Kassambara, 2020) were used to create figures.

The hormone treatments were considered two‐level factors (CORT yes/no and TH yes/no). CORT, TH, and their interaction CORT*TH were fixed effects in all the models examining the effects of hormone treatment. Model covariates were selected based on biological hypotheses. Non‐significant interactions were removed to avoid overfitting in all of the models.

For modeling breath rate at 14 days post‐hatch, the model included the following confounding covariates that were known to explain variation in nestling development and growth rate: wing length (proxy of individual structural size), brood size at 2 days post‐hatch (proxy of parental condition and nestling environment), and hatching date (proxy of parental condition and food availability). Breath rate models included nest ID as a random effect to account for non‐independence between individuals from the same nest. As only 37 out of the 83 individuals in the breath rate analysis were molecularly sexed, including sex would have decreased out sample size significantly, and therefore, we did not include sex in this model. We ensured that sex did not have a significant effect on breath rates in the subset of 37 sexed individuals (*F*_1,29.7_ = 0.47, *p* = 0.50).

For modeling the longitudinal DNA methylation measurements, the fixed effects, in addition to the hormone treatments, were sex, age (categorical variable: 7 or 14 days after hatching, or juvenile), the interaction between age and treatment (since the hormonal treatment may have distinct effects at different developmental stages), CpG‐site identity, and the interaction between CpG site and hormonal treatment (since methylation at different genomic positions may have different consequences on gene expression, projecting into the phenotype). Sex was also included in the model as a fixed effect since gene expression and DNA methylation were hypothesized to possibly have sex‐specific patterns (Nätt et al., 2014; Siller Wilks et al., 2023) and all the individuals were sexed. Due to a relatively small sample size (96 samples from 39 individuals) and many levels of CpG‐site identity consuming the degrees of freedom, we did not add other environmental covariates or non‐significant interactions to the model at the expense of overfitting. Random effects in the DNA methylation model were sample ID (12 and 4 CpG sites from the same sample for *NR3C1* and *THRB*, respectively), and individual ID (2 or 3 longitudinal samples from the same individual).

For modeling gene expression measured only at 14 days post‐hatch, the response variable was the log_2_ transformed relative gene expression. We included sex (as all individuals were sexed) as a fixed effect in the model in addition to the hormone treatments. As for the breath rate analysis, we included wing length, brood size at day 2, and hatching date as covariates in the model. The gene expression analysis did not include random effects since there were no repeated measures as only one individual at a single time point was included in this analysis as well as methylation analysis.

Furthermore, we tested for potential associations between breath rates, methylation, and gene expression. Gene promoter methylation was hypothesized to correlate negatively with gene expression (Bird, 2002). For both genes, the association between mean methylation per sample on their respective log_2_ transformed relative gene expression, and the association between log_2_ transformed relative gene expression on breath rates was examined with Pearson's correlation at 14 days post‐hatch. Additionally, we analyzed the associations between methylation levels of individual sites and gene expression, as individual sites may drive differences in expression (Jimeno et al., 2019).

## 3 RESULTS

### 3.1 Breath rate

**FIGURE 1:**
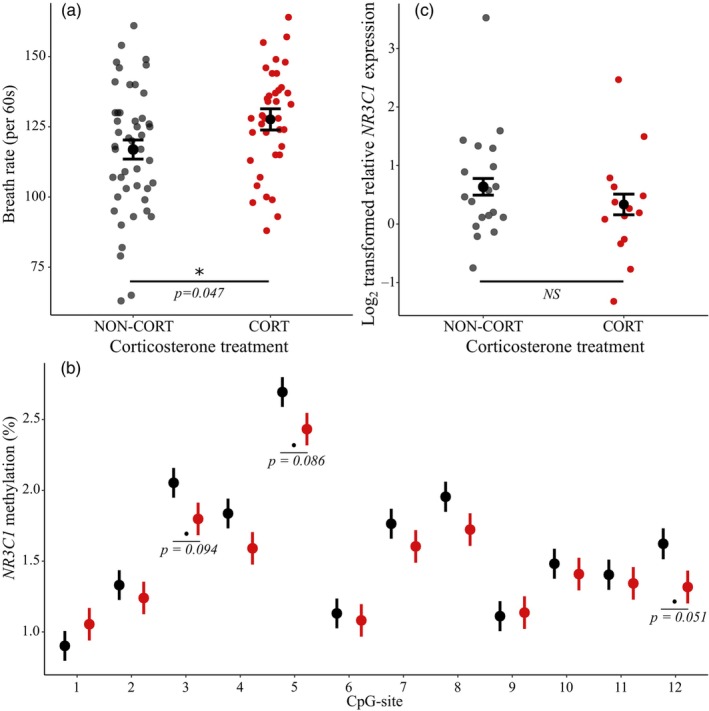
Effects of prenatal corticosterone manipulation on (a): breath rate 14 days after hatching (per 60s), (b): DNA methylation (%) at the quantified 12 CpG sites of *NR3C1* promoter region (black = non‐CORT, red = CORT) since a significant CORT*Site interaction was detected, and (c): glucocorticoid receptor *NR3C1* relative gene expression in blood cells. Estimated marginal means and standard errors are given (a–c), with the raw data (a,b). *p*‐values for the effect of corticosterone from type III ANOVA (a,b) and site‐specific post‐hoc comparison with Tukey's test (c) are also shown for *p* < 0.10.

Prenatal corticosterone treatment significantly increased breath rate during handling 14 days after hatching (Figure 1a, Table 5, effect size = 0.55). Neither prenatal thyroid hormone nor the interaction between prenatal corticosterone and thyroid hormones had a significant effect on breath rates (Table 5). While brood size and hatching date were not significantly associated with breath rate, individuals with longer wings (a proxy of individual size) had a marginally higher breath rate (Table 5, estimate **±** SE = 1.60 **±** 0.89, although non‐significant). The nest identity accounted for 11.6% of the variance in breath rate, which was not significant (Table 5, Supplementary Table S2).

**TABLE 5:** General linear (mixed) model explaining variation in breath rate, DNA methylation, and gene expression.

	Response variable				
	Breath rate (per 60 s)	DNA methylation (%)		Gene expression (log2 normalized)	
		NR3C1	THRB	NR3C1	THRA
Effect	*F*(ndf, ddf), *p*	*F*(ndf, ddf), *p*	*F*(ndf, ddf), *p*	*F*(ndf, ddf), *p*	*F*(ndf, ddf), *p*
CORT	4.29(1,30.1), **0.047**	1.08(1,34.1), 0.31	2.03(1,34.7), 0.16	1.80(1), 0.19	0.04(1), 0.84
TH	0.03(1,28.5), 0.87	0.12(1,34.4), 0.73	1.32(1,34.9), 0.26	0.01(1), 0.94	0.09(1), 0.76
Wing length	3.22(1,71.3), 0.077			26.28(1), **<0.001**	3.18(1), 0.086
Brood size D2	0.11(1,31.8), 0.95			1.41(1), 0.25	0.07(1), 0.79
Hatching date	0.29(1,37.6), 0.60			0.60(1), 0.44	1.10(1), 0.30
Sex		1.99(1,34.4), 0.17	1.71(1,34.9), 0.20	0.00(1), 0.99	0.07(1), 0.79
Site		99.04(11,1004.56), **<0.001**	118.6(3274.6), < **0.001**		
Age		0.24(2,59.9), 0.79	2.58(2,56.4), 0.085		
Site*CORT		2.39(11,1004.56), **0.006**	[1.84(3268.5), 0.14]		
Age*CORT		[0.12(02,55.8), 0.89]	[0.67(2,52.2), 0.52]		
Age*TH		[0.19(2,55.9), 0.83]	[0.26(2,52.3), 0.77]		
CORT*TH	[1.62(1,28.7), 0.21]	[0.70(1,32.9), 0.41]	[0.31(1,33.7), 0.58]	[0.49(1), 0.49]	[0.25(1), 0.62]
Site*TH		[0.85(11,993.5), 0.59]	[1.23(3268.6), 0.30]		
	Random effects				
	Variance explained	Variance explained	Variance explained		
Nest	11.6%				
Individual		**21.8%**	**42.4%**		
Sample		**31.3%**	**22.8%**		
Residual	88.4%	46.9%	34.8%		

*Note*: The test statistics for the main effects were from the final models without interaction terms. The exception was the model for *NR3C1* DNA methylation, which contains a significant interaction effect between corticosterone (CORT) and CpG site. Thus, the test statistics for the main effect of CORT represent the contrast with non‐CORT at CpG site 1, and the main effect of CpG site represents the CpG‐site difference in the non‐CORT group, respectively. See Section 3.2 and Figure 1b for post‐hoc comparisons. For fixed effects, Type III ANOVA *F*‐statistics, associated degrees of freedom, and *p*‐values are presented. Mixed models (breath rate and DNA methylation) are fit by REML, and degrees of freedom are estimated with Kenward–Roger method. For random effects, the percentage of variation explained (VE), and a test of significance (likelihood ratio test, with χ^2^ (df) and *p*‐value) are provided (see Supplementary Table S2). Significant effects are marked in bold. Brackets [] indicate non‐significant interaction terms removed from the final model.

### 3.2 DNA methylation

**FIGURE 2:**
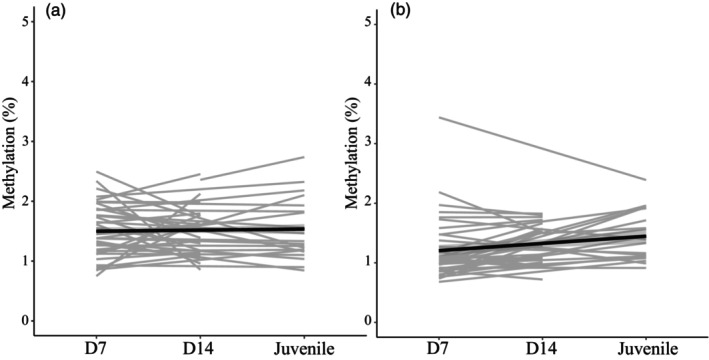
(a) Average methylation percentages pooled across different treatment groups at different ages for glucocorticoid receptor gene *NR3C1*. (b) Average methylation percentages pooled across different treatment groups at different ages for thyroid hormone receptor gene *THRΒ*. Black lines represent changes in methylation percentage in the overall mean (across all samples), and the grey lines represent changes in methylation percentage (averaged over all CpG sites) for each individual across ages.

Distinct CpG sites differed in their methylation value, with site explaining significant variation in DNA methylation for both *NR3C1* and *THRB* (Table 5, Figure 1b). Prenatal corticosterone treatment had CpG‐site‐specific effects on *NR3C1* promoter methylation (Figure 1b, Table 5, significant CORT*CpG site interaction). Tukey's post‐hoc comparisons revealed that for CpG sites 3, 5, and 12 of the target region, prenatal corticosterone decreased DNA methylation coming close to significance (all *p* < 0.094, Figure 1b, effect size = CpG site 3: −0.59, CpG site 5: −0.61, CpG site 12: −0.71). Thyroid hormone treatment, age (Figure 2a), sex, or the interactions between CORT and TH, age, and hormonal treatment, as well as CpG site and TH had no significant effect on DNA methylation at the *NR3C1* promoter region. A significant amount of variance (conditional on fixed effects) was explained by sample identity (12 CpG sites from the same sample, 31.3%) and individual identity (2 or 3 longitudinal samples from the same individual, 21.8%) (Table 5).

For *THRB*, DNA methylation tended to vary with age (p = 0.085, Table 5, Figure 2b, estimate **±** SE = Age D7: −0.24 **±** 0.11, Age D14: −0.17 **±** 0.11 compared to juveniles). Thyroid hormone treatment, sex, or the interactions between CORT and TH, age and hormonal treatment, CORT and CpG site, or TH and CpG site did not significantly affect *THRB* methylation (Table 5). Individual identity and sample identity explained a significant amount of variance in *THRB* methylation, 22.8% and 42.4%, respectively (Supplementary Table S2).

### 3.3 Gene expression

Neither corticosterone (Figure 1c, effect size = *NR3C1*: −0.50, *THRA*: 0.08) nor thyroid hormone treatment had a significant effect on gene expression 14 days post‐hatch (Table 5). For *NR3C1*, wing length was significantly negatively associated with gene expression (Table 5, estimate **±** SE = −0.14 **±** 0.028), whereas sex, brood size, and hatching date had no significant effect on gene expression.

For *THRA*, only wing length tended to have a significant negative relationship with gene expression (estimate **±** SE = −0.093 **±** 0.052, although not significant), whereas no other variable exhibited a significant relationship with *THRA* gene expression (Table 5).

### 3.4 Correlations

**FIGURE 3:**
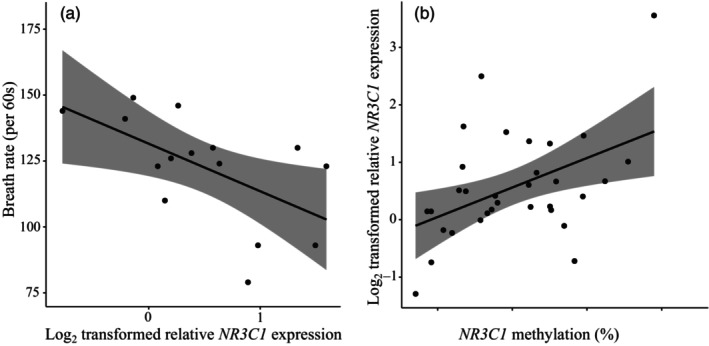
(a) Correlation between breath rate (per 60s) and *NR3C1* gene expression. (b) Correlation between *NR3C1* relative gene expression and putative promoter mean methylation (%). Regression line and 95% confidence limits are given with the raw data.

While analyzing possible relationships between the variables of interest at day 14 post‐hatch, we found *NR3C1* gene expression to have a significant negative bivariate correlation with breath rates (Figure 3A, *r* = −0.58, *p* = 0.022). Furthermore, *NR3C1* sample mean methylation had a positive bivariate correlation with its gene expression (Figure 3b, *r =* 0.46, *p* = 0.007). When analyzing the sites individually, seven sites were significantly positively correlated with *NR3C1* gene expression, three were non‐significant with positive estimates, and two were non‐significant with negative estimates (Supplementary Figure S2). *THRA* expression showed no significant relationships with breath rates or *THRB* methylation (all *p*‐values >0.05).

## 4 DISCUSSION

Elevation of prenatal corticosterone increased breath rates in great tits 14 days after hatching. Prenatal corticosterone also tended to decrease GR gene *NR3C1* promoter methylation in three of the 12 studied CpG sites, but no age‐specific patterns were observed. Prenatal corticosterone had no significant effect on gene expression of GR (although effect size was of similar magnitude as for breath rate or methylation) or THR, while GR gene expression was negatively associated with breath rates and showed a strong association with both wing length and hatching date. Elevation of prenatal THs did not influence breath rate, THR methylation, or gene expression.

### 4.1 The effects of prenatal hormones on breath rates, DNA methylation, and gene expression

#### 4.1.1 Prenatal corticosterone treatment

In line with our hypothesis and previous studies (e.g., Tilgar et al., 2016, *reviewed in* Thayer et al., 2018), prenatal supplementation of great tit eggs with corticosterone significantly increased breath rate, a measure of metabolic stress response, at 14 days after hatching. Increased breath rate may result from the facilitation of the sympathetic‐adrenal‐medullary response by glucocorticoids (Ouyang et al., 2021). We were unfortunately not able to assess baseline or stress‐induced glucocorticoid levels in this study. The evidence for prenatal GC exposure to affect circulating GC levels is relatively equivocal since *in ovo* corticosterone treatment has been found to both increase (Ahmed et al., 2014; Freire et al., 2006; Haussmann et al., 2012; Marasco et al., 2012) and decrease (Hayward et al., 2006; Love & Williams, 2008; Tilgar et al., 2016) baseline as well as stress‐induced corticosterone levels. The discrepancy in previous studies shows that HPA axis regulation may be subject to maternal corticosterone, affecting stress response, yet there may be other biological mechanisms involved and the effects are likely timing‐, context‐, and dose‐dependent. Yet, the current study shows that pre‐incubation corticosterone can increase offspring breath rate during handling in great tits, therefore highlighting the role of maternal effects in shaping offspring metabolic response to stress.

The molecular mechanism underlying the observed prenatal hormonal effects on postnatal breath rates may be related to epigenetic changes since our corticosterone treatment also had CpG‐site‐specific effects, mainly decreasing DNA methylation in the putative promoter area of the glucocorticoid receptor (GR, *NR3C1*) gene. In line with our results, Ruiz‐Raya et al. (2023) found prenatal exposure to alarm calls to reduce GR promoter methylation in yellow‐legged gull (*Larus michahellis*) blood cells. However, these results on the site‐specific decreased methylation at GR gene promoter after corticosterone treatment are not in agreement with our primary hypothesis nor with the few previous studies investigating prenatal and early life stress, and GR methylation. In domestic chickens, a high concentration of corticosterone injected into eggs around mid‐incubation increased hypothalamic GR methylation (Ahmed et al., 2014). Bockmühl et al. (2015) found that postnatal early‐life stress in mice increased hypothalamic CpG island shore methylation at certain CpG sites at GR. Jimeno et al. (2019) observed an increase in blood GR promoter methylation resulting from postnatal early‐life adversity. Azar and Booij (2022) reviewed prenatal maternal stress to increase offspring peripheral DNA methylation of the GR gene. Yet, to our knowledge, this is the first study assessing the effects of pre‐incubation corticosterone injection on the methylation status of this gene in blood cells.

Corticosterone treatment did not influence GR expression significantly, though the effect size was of the same magnitude as for the effects of corticosterone treatment on breath rates and GR methylation (~0.5). It is possible that prenatal corticosterone did not influence GR expression, or that the sample size in the current study was too low to detect a significant effect. Previous studies have found prenatal stress to alter GR expression, yet the direction of the change has been equivocal across tissues and species (Cottrell & Seckl, 2009; Kapoor et al., 2006; Ruiz‐Raya et al., 2023; Zimmer et al., 2017). As GR methylation and gene expression were positively correlated in our study, it is possible that prenatal corticosterone causes site‐specific GR methylation alterations that would have consequences on gene expression. Indeed, it seems that in our data, seven of the 12 CpG sites of the GR gene were significantly positively correlated with its expression, while for the rest of the sites, there was no significant correlation. Two of these sites, CpGs 3 and 5, were also sites where prenatal corticosterone had a close to significant, decreasing effect on DNA methylation. This complex, possibly activating role of methylation at some CpG sites has some support from previous literature: Bockmühl et al. (2015) found early‐life stress to increase hypothalamic GR expression by site‐specific CpG island shore hypermethylation. In yellow‐legged gull blood cells, Ruiz‐Raya et al. (2023) found no correlation between GR expression and average promoter methylation levels, or CpG‐site‐specific promoter methylation, yet they did find GR expression to associate with principal component 2 derived from methylation data, which further supports the multifaceted role of promoter methylation in transcriptional regulation. Yet, the positive relationship between methylation at regulatory CpGs and gene expression contrasts the canonical view of the suppressive role of promoter methylation (Bird, 2002). Further, our functional conclusions are limited since prenatal hormone treatment might have influenced DNA methylation and gene expression more strongly in other tissues, for instance, directly within the HPA axis. Stress‐related methylation changes in peripheral tissues such as blood have been found to reflect methylation patterns also in central nervous system in mammals (Ewald et al., 2014; Palma‐Gudiel et al., 2015), but there is a lack of data on this topic in avian species so far. As our results on decreased DNA methylation after corticosterone treatment were measured in blood, and changes in the promoter region of GR did not directly translate to significant differences in GR expression, the molecular mechanisms remain to be fully elucidated.

#### 4.1.2 Prenatal thyroid hormone treatment

Prenatal TH supplementation of great tit eggs did not significantly alter breath rates, GR or THRB DNA methylation status, or gene expression at the GR or THRA genes. There are several plausible, mutually non‐exclusive, explanations for this. First, it could be that prenatal THs do not have a strong effect on offspring hormonal signaling and stress‐related phenotype. The lack of effects of prenatal TH supplementation is in line with results from the same experimental birds, which revealed no significant effect of thyroid hormones on growth or mitochondrial metabolism (Cossin‐Sevrin et al., 2022). This suggests that experimental corticosterone may have stronger leverage on offspring stress‐related phenotype than TH with this experimental setup. Second, the genes we analyzed might not be targets of prenatal THs and they may exert their actions on other biological pathways (Vitousek et al., 2019). Third, it could be that the effects of the hormone treatments could have been observed in other genomic locations, especially as our methylation analysis in the putative *THRB* promoter only included four CpG sites after quality filtering. Fourth, the effects of maternal hormones are dependent on the expression of transport molecules, cell membrane transporters, and deiodinases facilitating the conversion of TH between the inactive and active forms (McNabb & Wilson, 1997; Ruuskanen & Hsu, 2018). In chickens, a high level of expression of deiodinase (DIO) type 3 by the yolk sac membrane was found since embryonic day 5 (Too et al., 2017), which might have some function in deactivating excessive THs. In passerines, Ruuskanen et al. (2022) also found early‐stage embryos to express DIO2, DIO3, THRA, THRB, and monocarboxyl membrane transporter MCT8, suggesting that altricial embryos could be able to modulate the effects of egg TH during embryonic development. Fifth, prenatal TH elevation may have tissue‐specific effects on gene methylation and expression, such as in the brain, but not in blood cells (Bockmühl et al., 2015; Lattin et al., 2015; McCormick et al., 2000, but see support for between‐tissues correlations in Daskalakis et al., 2014). Yet, even if blood and brain hormone receptors are not tightly correlated, information on blood levels may provide valuable functional information, for example, when sampling other tissues is not feasible (Jimeno & Zimmer, 2022). Sixth, prenatal THs work in synergy with hormones that were not included in this study, for example, Wang et al. (2007) found that oral dosing of thyroid hormone (T3) together with growth hormone injections had synergist effects on body fat and hepatic gene expression of juvenile chickens.

### 4.2 Patterns of DNA methylation

The overall methylation percentages for both genes, glucocorticoid receptor and thyroid hormone receptor, were generally low (median <2%). These results corroborate with previous findings from birds where CpG‐dense promoters and transcription start sites are less methylated specifically for the gene coding for GR (yellow‐legged gulls; Ruiz‐Raya et al., 2023), as well as for genome‐wide patterns (great tits: Derks et al., 2016; Laine et al., 2016). Derks et al. (2016) found that great tit transcription start sites in the brain and blood are generally lowly methylated in the tissues in which they are anticipated to be expressed compared to tissues where they are not expressed. Both genes of interest, glucocorticoid receptor GR and thyroid hormone receptor THRΒ, exhibited statistically significant differences between the methylation percentages of individual CpG sites. These results suggest that certain CpG‐site methylation may be more important in the regulation of gene expression rather than the average methylation percentage of a certain CpG island. Indeed, it has been shown that even within promoters, the entire sequence may not be methylated in the same way and short sequences may have distinct methylation patterns depending on which transcription factors bind which sites (Tohgi et al., 1999).

For GR, a large proportion of variance in methylation percentages, conditional on fixed effects, was explained by sample identity (i.e., 31.3%), implying consistency in the methylation percentage between different CpG sites of the same sample, potentially due to co‐methylation over neighboring CpG sites (Eckhardt et al., 2006; Jimeno et al., 2019). This may also be due to sample‐specific blood cell type composition, which may vary both within and between individuals, although the vast majority of avian blood DNA comes from nucleated red blood cells (Husby, 2020). More importantly, a significant amount of variance (i.e., 22.0%) was explained by individual identity, which demonstrates consistent inter‐individual differences in methylation through time. For THRΒ, the largest proportion of variance was explained by individual identity (42.4%), but sample identity also explained a substantial part of the variance (22.8%). The relatively high within‐individual consistency in methylation percentages revealed by our longitudinal approach suggests that methylation patterns for different individuals had persisted from 7 days of age to juvenility. To the best of our knowledge, this is the first evidence of such within‐individual consistency in methylation pattern in wild animals, but parallels what has been described in human (Di Sante et al., 2018). This indicates a relatively robust and consistent methylation in the analyzed regions across time. In contrast, Siller Wilks et al. (2023) found significant differences in house sparrow (*Passer domesticus*) methylation between days 2 and 10 in HPA function‐related genes in blood cells. Regarding developmental effects on DNA methylation resulting from early‐life stress, Bockmühl et al. (2015) found increased methylation in older individuals (10 days vs. 6 weeks vs. 3 months old in a cross‐sectional design) in certain CpG sites in the shore region of a CpG‐island upstream from GR in the hypothalamus of mice that had been exposed to early‐life stress. Early‐life stress induced an increase in the overall methylation of this CpG‐island shore that was only observed at a later age (Bockmühl et al., 2015). In turn, Marasco et al. (2012) injected corticosterone into the eggs of Japanese quail and found a hyper‐regulated HPA response as elevated circulating corticosterone levels during acute stress at 64 days after hatching, but not at 22 days after hatching. In the same species, another study using similar doses did not find overall effect on circulating CORT levels, but prenatally CORT‐supplemented group had faster decrease in CORT levels after the peak levels at 43 days after hatching (Zimmer et al., 2013). The results of these previous studies suggest that the effects of prenatal hormonal treatments might be more evident after growth completion. Consequently, the time interval between prenatal hormonal injection and post‐hatching days 7 and 14 was maybe too short to detect possible differences in methylation percentages resulting from prenatal hormonal treatment. The postnatal period is also the time when HPA axis is maturing in altricial birds (e.g., Wada et al., 2007), and therefore, effects of prenatal hormones on HPA axis may be more evident later in life. However, we found no significant impact in juveniles (approximately 4 months old) either, but the number of juveniles for each treatment group was only five (four for THRΒ CORT group), which may have been a sample size too small to detect moderate treatment differences in methylation percentages.

### 4.3 Patterns of gene expression

There was a negative relationship between body size (i.e., wing length) and GR gene expression, with larger individuals having lower GR expression levels. Nutritional or developmental stages may explain the relationship between body size and GR expression. Food insecurity and malnutrition have been found to stimulate stress and increase cortisol levels in humans (Freitas et al., 2018; Sawaya, 2006), which in turn could influence stress hormone receptor expression (Cottrell & Seckl, 2009). Alternatively, it might be possible that a lower GC signaling through decreased GR expression enhances growth by favoring energy allocation into anabolic processes, which requires further investigation. Establishing the potential directional causal link between GR and growth will, however, require experimental work directly manipulating signaling through GR.

In contrast to GR expression, THRΑ expression did not significantly correlate with any of the studied biological and ecological covariates (wing length, hatching date, brood size, breath rate). A variety of factors could alter thyroid hormone levels of the nestling's mother's plasma, such as food and iodine availability, endocrine‐disrupting molecules, stress, and therefore indirectly also pathogens and intra‐ and interspecies interactions (Ruuskanen & Hsu, 2018). Therefore, a robust regulation in TH receptor expression may be adaptive to protect the developing individuals from environmental and/or physiological variation both pre‐ and post‐hatching.

## 5 CONCLUSIONS

This study supports the view that maternal corticosterone may influence offspring phenotype, possibly via epigenetic alterations. Yet, the functional, causal link between prenatal hormones, HPA‐related epigenetics, and phenotypic changes remains elusive and is an important avenue for coming research. We encourage future studies to analyze whole‐genome methylation patterns and transcriptomic profiles to elucidate the pathways linking prenatal hormonal exposure and postnatal HPA response. Furthermore, the possible adaptive role of phenotypic changes resulting from prenatal hormones needs to be studied by assessing long‐term fitness consequences of these effects across different environments.

## CONFLICT OF INTEREST STATEMENT

The authors declare no conflicts of interest.

## Supporting information


Supplementary Table 1.

## Data Availability

All data and R scripts are archived and available in Figshare (DOI: 10.6084/m9.figshare.22153001; Hukkanen et al., 2023).
