# Supplementary material for: From maternal glucocorticoid and thyroid hormones to epigenetic regulation of offspring gene expression: An experimental study in a wild bird species
Source: Evol Appl. 2023 Oct 3;16(10):1753–69. doi: 10.1111/eva.13598 (PMC10660793; doi:10.1111/eva.13598)
Supplement: Supplementary file 1 — Supplementary Table 1. [file EVA-16-1753-s001.docx]

Electronic supplementary materials of ´From maternal glucocorticoid and thyroid hormones to epigenetic regulation of offspring gene expression: an experimental study in a wild bird species`

Supplementary Table 1. Sex ratios: number of individuals (F:M) included in the analysis after quality filtering for each gene (*NR3C1*=glucocorticoid receptor; *THRB*=thyroid hormone receptor β), treatment group (CO=control; CORT=corticosterone; CORT + TH=corticosterone and thyroid hormone; TH=thyroid hormone) and age (DAH=days after hatching).

| NR3C1 | | | | | |
| --- | --- | --- | --- | --- | --- |
| Age | CO | CORT | CORT + TH | TH | Total |
| 7 DAH | 10 (4:6) | 9 (2:7) | 9 (1:8) | 10 (4:6) | 38 (11:27) |
| 14 DAH | 10 (4:6) | 10 (3:7) | 8 (1:7) | 10 (4:6) | 38 (12:26) |
| Juvenile | 5 (2:3) | 5 (2:3) | 5 (1:4) | 5 (1:4) | 20 (6:14) |
| Total | 25 (10:15) | 24 (7:17) | 22 (3:19) | 25 (9:16) | 96 (29:67:2) |
| THRB | | | | | |
| Age | CO | CORT | CORT + TH | TH | Total |
| 7 DAH | 10 (4:6) | 10 (3:7) | 8 (1:7) | 10 (4:6) | 39 (12:26) |
| 14 DAH | 10 (4:6) | 10 (3:7) | 7 (0:7) | 10 (4:6) | 38 (11:26) |
| Juvenile | 5 (2:3) | 4 (1:3) | 5 (1:4) | 5 (1:4) | 19 (5:14) |
| Total | 25 (10:15) | 24 (7:17) | 22 (2:18) | 25 (9:16) | 94 (28:66) |

Supplementary Table 2. Estimated proportion of variance (%Var) explained by random effects for all models. The proportion of explained variance was calculated as the estimated variance for the random factor in question divided by the total variance. Significance tests were performed by log-likelihood ratio test between models with different random structures. Significance is marked in bold.

| Breath rate | | | | | | | | | | |
| --- | --- | --- | --- | --- | --- | --- | --- | --- | --- | --- |
| Random effect(s) | %Var | Model | Parameters | AIC | Log likelihood | Deviance | Test | χ2 | Δdf | p |
| None |  | 1 | 7 | 745.99 | -366.00 | 731.99 |  |  |  |  |
| Nest | 11.6 | 2 | 8 | 747.76 | -365.88 | 731.76 | 1 vs 2 | 0.2284 | 1 | 0.63 |
| Residual | 88.4 |  |  |  |  |  |  |  |  |  |
| DNA methylation: NR3C1 | | | | | | | | | | |
| Random effect(s) | %Var | Model | Parameters | AIC | Log likelihood | Deviance | Test | χ2 | Δdf | p |
| Sample | 21.8 | 1 | 30 | 1576.4 | -758.22 | 1516.4 | 1 vs 3 | 243.64 | 1 | **<0.001** |
| Individual | 31.3 | 2 | 30 | 1811.0 | -875.51 | 1751.0 | 2 vs 3 | 9.052 | 1 | **0.003** |
| Individual and sample |  | 3 | 31 | 1569.4 | -753.69 | 1507.4 |  |  |  |  |
| Residual | 46.9 |  |  |  |  |  |  |  |  |  |
| DNA methylation: THRB | | | | | | | | | | |
| Random effect(s) | %Var | Model | Parameters | AIC | Log likelihood | Deviance | Test | χ2 | Δdf | p |
| Sample | 22.8 | 1 | 11 | 537.43 | -257.71 | 515.43 | 1 vs 3 | 27.18 | 1 | **<0.001** |
| Individual | 42.4 | 2 | 11 | 557.62 | -267.81 | 535.62 | 2 vs 3 | 47.37 | 1 | **<0.001** |
| Individual and sample |  | 3 | 12 | 512.25 | -244.13 | 488.25 |  |  |  |  |
| Residual | 34.8 |  |  |  |  |  |  |  |  |  |


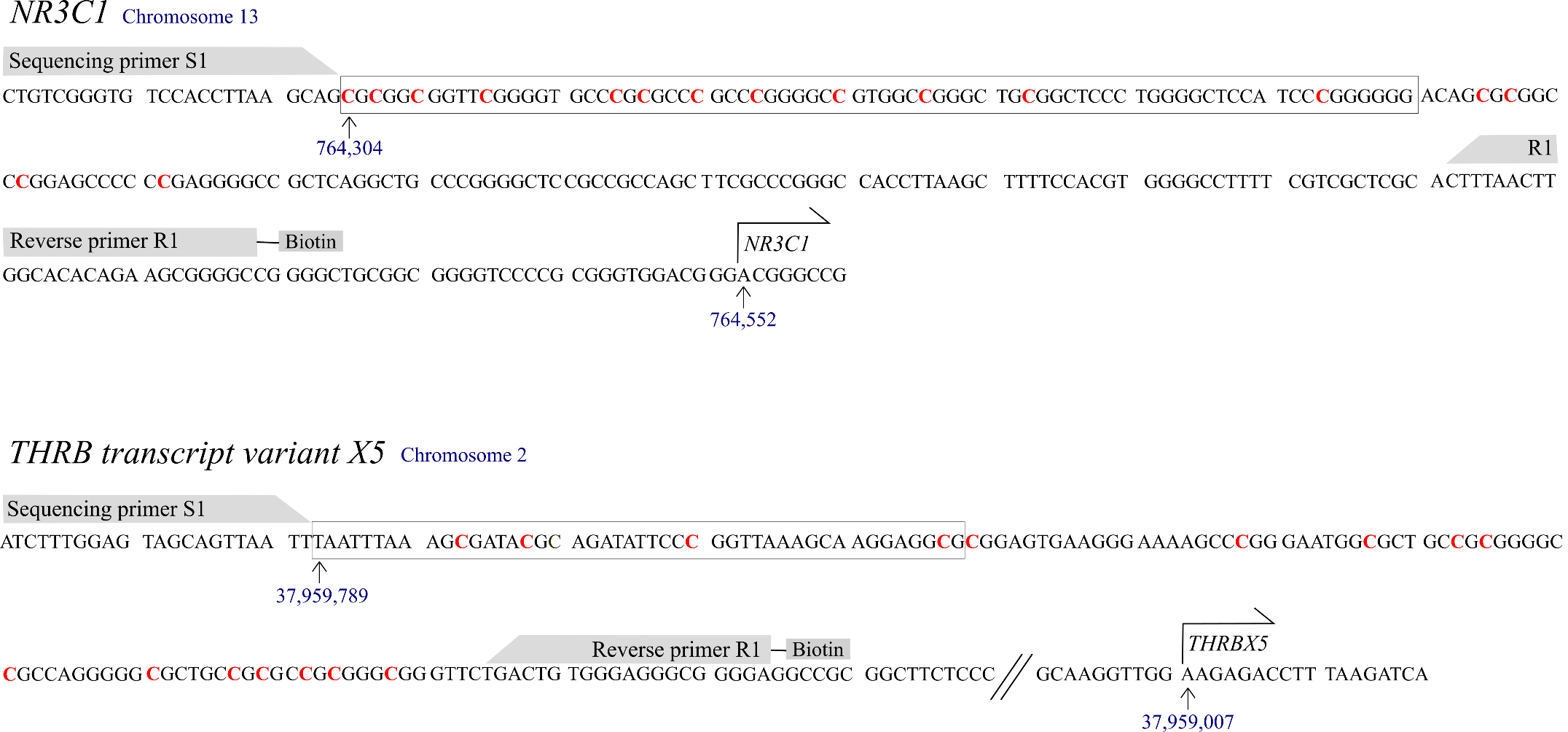
Supplementary Figure 1. Sequence analyzed in pyrosequencing. Primer regions are marked with solid grey boxes. All analyzed 16 CpG-sites are marked with red. Sequence analyzed after quality filtering is lined in black. Gene start is marked with arrow and gene name. Genomic locations with respect to the respective chromosome are marked below the sequences in dark blue.


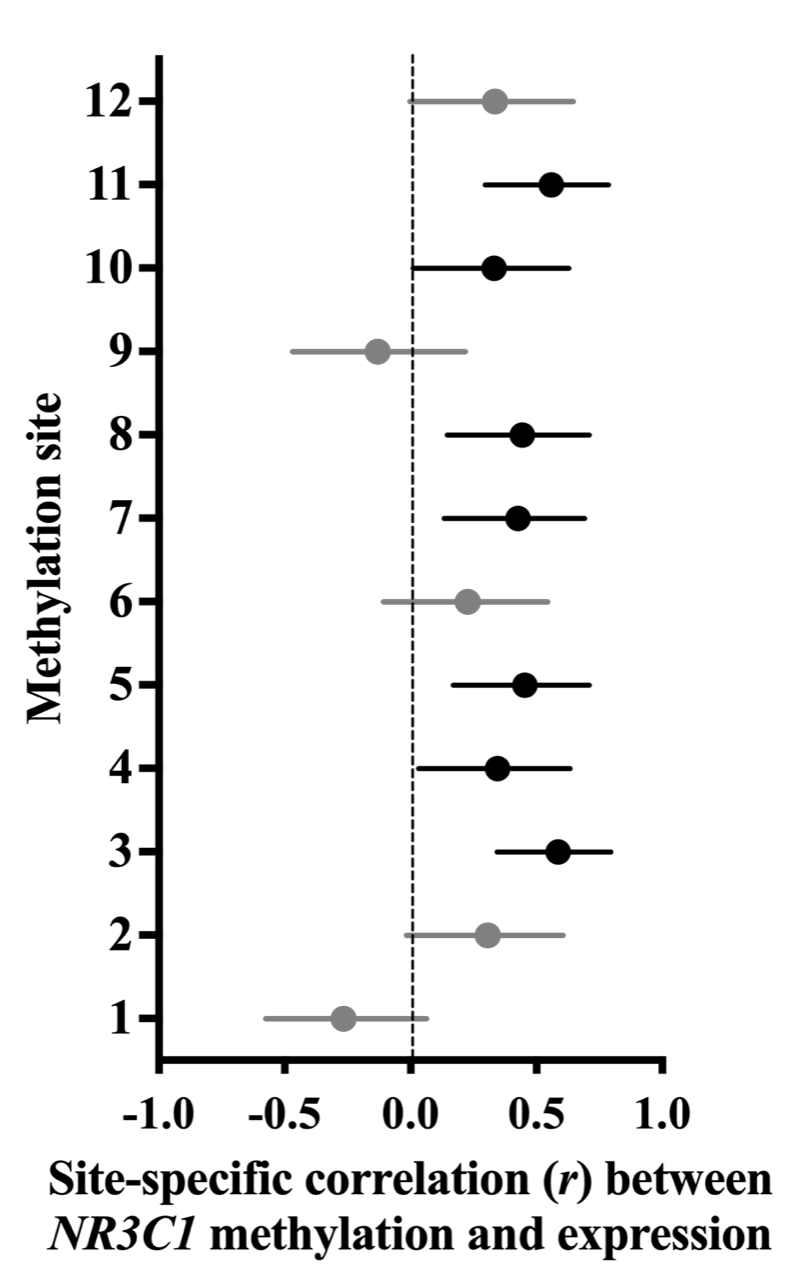


Supplementary Figure 2. Site-specific correlation between DNA methylation and gene expression for NR3C1. Pearson correlation coefficients are given with 95% confidence intervals.
